# Surgical Results of Retrograde Mastoidectomy with Primary Reconstruction of the Ear Canal and Mastoid Cavity

**DOI:** 10.1155/2015/517035

**Published:** 2015-03-15

**Authors:** Chao-Yin Kuo, Bor-Rong Huang, Hsin-Chien Chen, Cheng-Ping Shih, Wei-Kang Chang, Yang-Lien Tsai, Yuan-Yung Lin, Wan-Chun Tsai, Chih-Hung Wang

**Affiliations:** ^1^Department of Otolaryngology-Head and Neck Surgery, Tri-Service General Hospital, National Defense Medical Center, No. 325, Section 2, Cheng-Kung Road, Taipei 114, Taiwan; ^2^School of Pharmacy, Kaohsiung Medical University, No. 100, Shih-Chuan 1st Road, Kaohsiung City 80708, Taiwan; ^3^Department of Medical Administration, Kaohsiung Armed Force General Hospital, No. 2, Zhongzheng 1st Road, Kaohsiung City 80284, Taiwan; ^4^Graduate Institute of Medical Sciences, National Defense Medical Center, No. 161, Section 6, Minquan East Road, Taipei 114, Taiwan

## Abstract

The aim of this study was to retrospectively review the long-term hearing results and the impact of mastoid exclusion/obliteration in patients with cholesteatoma (102 ears) who underwent retrograde tympanomastoidectomy and in whom bone chips/paté were applied as the sole materials during the procedure. In 79 ears, this was combined with ossiculoplasty in a single-stage procedure. In >71% of ears, the results of audiometric testing were monitored for more than 2 years. The results suggested there was a significant gain in hearing following surgery, with respect to the postoperative change in both air-conduction thresholds and air-bone gaps (*P* < 0.001). Linear regression analyses of pure-tone averages at different frequencies, before and after surgery, demonstrated that patients benefitted from a postoperative hearing gain at low and middle frequencies, but their hearing often deteriorated at frequencies of 8000 Hz. As for the impact of the type of tympanoplasty on hearing outcomes, type III-interposition markedly increased hearing gain. The overall rate of postoperative adverse events was 8.8%. We conclude that reconstruction of the ear canal and mastoid via mastoid exclusion/obliteration using bone chips/paté can be considered as an alternative procedure following retrograde mastoidectomy. It gives excellent surgical results and has fewer postoperative adverse events.

## 1. Introduction

For a long time, the primary objective in the surgical management of cholesteatoma has been the eradication of disease, as well as ensuring a dry and safe ear. Hearing preservation or restoration is also worth considering but is often treated as a secondary goal. Controversies in surgical management of cholesteatoma include the choice in surgical approach, that is, canal wall down (CWD) or canal wall up (CWU), and the need for staged operations [1, 2].

Although cholesteatoma can develop in up to 10% of people with chronic suppurative otitis media, its actual pathogenesis is still unknown [3]. In past decades, both the incidence and the referral rate of cholesteatoma at our center have decreased. This is in accordance with the findings of a study from Denmark that observed a statistically significant decline in the incidence rate of cholesteatoma from 1977 to 2007 [4]. In addition, the extensiveness and aggressiveness of cholesteatoma have also abated compared to two decades ago.

In 1995, the National Health Insurance System was introduced in Taiwan, which markedly influenced people's behavior in seeking medical advice. As a consequence, more patients with cholesteatoma are presenting with earlier stages of the disease and fewer symptoms of hearing destruction. Therefore, as the clinical characteristics of extensive cholesteatoma have changed, the demand for hearing preservation has increased. This makes hearing preservation and restoration as important as disease eradication when considering surgical removal of cholesteatoma.

CWD mastoidectomy allows for better visualization, greater assurance of cholesteatoma eradication, and a lower recidivism rate than CWU, but at the expense of the need for life-long mastoid care. On the other hand, CWU mastoidectomy is not associated with mastoid bowl problems and allows for good hearing outcomes. However, it is associated with a higher recidivism rate than CWD owing to poor visualization, and an increased likelihood of the postoperative formation of a retraction pocket. Retrograde tympanomastoidectomy combines the virtues of both CWD and CWU procedures by the partial or entire removal of the posterior bony canal wall, depending on disease extent [5, 6]. Most importantly, such approach helps to create the smallest cavity necessary to remove all the cholesteatomas so as to preserve more external auditory canal (EAC) bone to facilitate reconstruction.

Early attempts to carry out mastoid obliteration mostly used local flaps [7, 8]. Since then, many other materials have been introduced for this procedure, such as autologous bone graft [9], bone paté [10], cartilage [11], hydroxyapatite cement/granules [12–14], and composite multifractured osteoperiosteal flaps [15]. The purpose of mastoid obliteration in several modifications may involve the promotion of healing in mastoidectomy defects, preservation of hearing, and elimination of cavity-related problems such as infection, recurrent retraction pockets, or cholesteatoma. In addition to offering mastoid obliteration to those enrolled in this study, we also offered another surgical technique—mastoid exclusion. This involves placing several pieces of bony plates and bone chips on the preserved canal wall and tegmen tympani, which not only isolates the mastoid cavity but also completes the reconstruction of the EAC defect in a one-stage surgical procedure.

This retrospective study aims to evaluate the impact of mastoid exclusion/obliteration in retrograde tympanomastoidectomy for patients with cholesteatoma, using bone chips/paté as the sole materials, and with an emphasis on its long-term postoperative audiometric outcomes.

## 2. Materials and Methods

### 2.1. Study Approval and Patients

This was a retrospective chart review study, evaluating the surgical outcome and pure-tone audiograms of patients with cholesteatoma, before and after surgical treatment. The research was approved by the Institutional Review Board of Tri-Service General Hospital, National Defense Medical Center, Taipei, Taiwan (Protocol number 2-103-05-139). Study subjects included patients with cholesteatoma who were treated at the Department of Otolaryngology-Head and Neck Surgery, Tri-Service General Hospital, Taipei, Taiwan, from January 2004 to October 2013. A total of 99 patients (102 ears) were enrolled in this study, all of whom underwent retrograde tympanomastoidectomy; in 79 ears, this was combined with ossiculoplasty in a single-stage procedure (Table 1).

The medical records of patients with cholesteatoma who underwent retrograde tympanomastoidectomy by the senior author (CHW), in conjunction with mastoid exclusion/obliteration surgery using bone chips/pate, were included in the study. Exclusion criteria were as follows: (1) diagnosis other than cholesteatoma, (2) the follow-up hearing test was conducted less than one year after surgery, and (3) there was a greater than 50% destruction of the EAC, which was reconstructed with a transposition of the muscle flaps or a CWD procedure. We performed this technique only in cases involving less than 50% destruction of the EAC following retrograde mastoidectomy, due to the feasibility of EAC reconstruction using bone chips/paté as the sole material in these instances. In the face of extensive cholesteatoma resulting in greater destruction of the EAC or CWD procedure, a meatally based musculoperiosteal flap was modified for mastoid obliteration in conjunction with the use of bone chips/paté, but such cases were excluded from this analysis.

We retrospectively reviewed each medical record to obtain the patient demographic, the surgical procedure they underwent, the materials used for ossicular chain reconstruction, the length of follow-up, associated complications, and hearing outcomes.

### 2.2. Hearing Outcome Measures

All patients underwent preoperative and postoperative pure tone audiometry. It was suggested that patients should complete hearing tests every 6 months after surgery for the first 2 years, and at yearly intervals thereafter. As mentioned in the exclusion criteria, we excluded individuals with audiometric data covering less than 1 year of postoperative follow-up. In addition, postoperative hearing outcomes were determined using the audiogram records at the latest follow-up. A four-frequency pure-tone average (PTA) was calculated from the average pure-tone audiometry at 500, 1000, 2000, and 4000 Hz. The audiometry results were reported according to the Committee on Hearing and Equilibrium guidelines, except for thresholds at 3000 Hz, which were substituted with thresholds at 4000 Hz [16]. Pure-tone audiometry thresholds at 500, 1000, 2000, and 4000 Hz via air (AC) and bone conduction (BC) were determined and the air-bone gaps (ABGs) were calculated. Postoperative BC values were used to calculate the postoperative ABG. Mean preoperative and postoperative AC threshold, ABGs, and improvements in AC and ABGs were recorded at each frequency (250, 500, 1000, 2000, 4000, and 8000 Hz).

The degree of hearing loss based on AC-PTA is defined by four levels [17], measured in decibels hearing level (dB HL): mild (≤40 dB HL), moderate (41–70 dB HL), severe (71–90 dB HL), and profound (≥91 dB HL).

### 2.3. Surgical Technique

#### 2.3.1. Retrograde Tympanomastoidectomy for Cholesteatoma Removal

**Figure 1:**
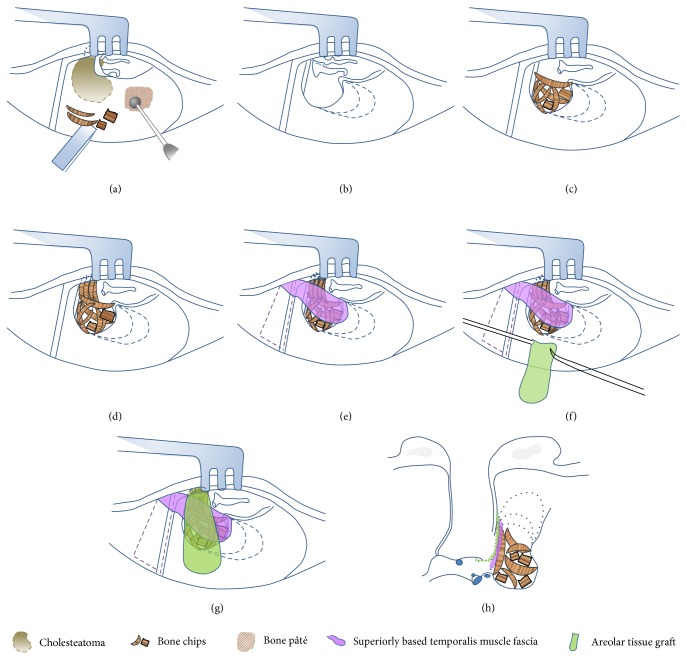
Schematic representation of the surgical procedure for mastoid exclusion. (a) Bone chips and paté are harvested with chisels and drill from the healthy mastoid cortex at the beginning of the mastoidectomy. (b) A retrograde mastoidectomy is performed to remove the cholesteatoma. Dotted lines indicate areas for further exposure to be achieved as needed. (c) The ear canal wall is reconstructed with several pieces of curved bony plates placed on the preserved canal wall and tegmen tympani. The isolated mastoid cavity is filled with bone chips. (d) Several pieces of bone chips are placed medially on the attic wall to obliterate the protympanum while the malleus head is removed. (e) Superiorly based temporalis muscle fascia is rotated to cover the underlying bone chips. (f, g) An areolar tissue graft overlaps the rotated fascia and is positioned under the eardrum remnant using the underlay technique. (h) Coronal view.

The procedure usually involves a postauricular incision and general anesthesia. In the early stages of the mastoidectomy, healthy bone chips and bone paté were harvested with chisels and drills, respectively, from an uninvolved area of the mastoid cortex (Figure 1(a)).

The retrograde mastoidectomy was initiated by drilling the ear canal and following the extent of the cholesteatoma until noninvolved cavity with healthy mucosa was reached. This approach led to the exposure of cavities of different sizes among individuals following the mastoidectomy (Figure 1(b)). In cases of atticoantral cholesteatoma, a complete removal of the cholesteatoma may require the sacrifice of parts of the ossicular chain.

#### 2.3.2. Reconstruction of the EAC

The ear canal wall was reconstructed with several pieces of curved bony plates placed on the preserved canal wall and tegmen tympani. The isolated mastoid cavity was filled with bone chips (Figure 1(c)). The bone chips and bone paté were harvested at the very beginning of the mastoidectomy, stored in gentamycin solution (80 mg/2 mL), and washed with normal saline before application. In accordance with the ossicular chain status, in cases in which the malleus head had been amputated, the protympanum and epitympanum were sealed off by medially placing several pieces of bone chips on the attic wall (Figure 1(d)). A superiorly based temporalis muscle fascia was rotated into the mastoid cavity to cover the underlying bone chips (Figure 1(e)). Then, using an underlay technique, an areolar tissue graft was taken and was overlapped with the rotated fascia to repair any defects in the tympanic membrane (Figures 1(f) and 1(g)).

**Figure 2:**
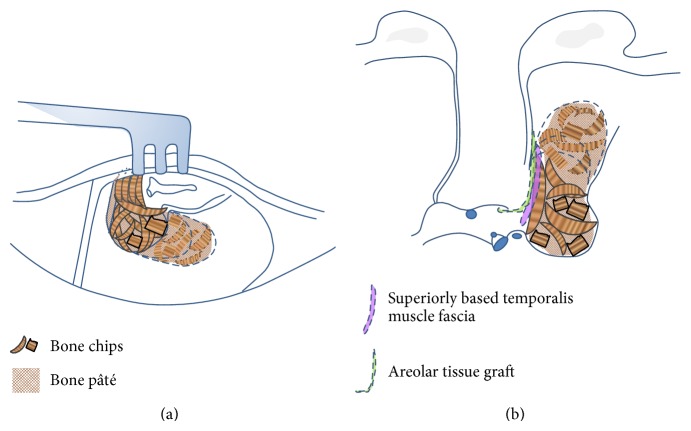
A schematic representation of the surgical procedure for mastoid obliteration. (a) The mastoid bowl (created as a result of retrograde mastoidectomy and indicated by dotted lines) is filled with a combination of bone chips and bone paté. (b) Coronal view.

By contrast, for cases of mastoid obliteration, the mastoid cavity was filled with bone chips and bone paté in combination with the covering of a superiorly based temporalis muscle fascia after mastoid air cells and the mucosa lining had been eradicated (Figure 2).

Drain set placement is not necessary after this operation. To clear up the infection, intravenously administered 1000/200 mg of amoxicillin/clavulanic acid three times a day, plus 80 mg of gentamycin two times a day are usually given in the first 3–5 days after the operation. This was followed by 500 mg of oral ciprofloxacin twice daily for 1 to 2 weeks.

#### 2.3.3. Reconstruction of Ossicular Chain

Patients whose ossicular chain had either been affected by cholesteatoma or had been removed during surgery were subject to an ossiculoplasty in a single-stage tympanomastoidectomy (79 ears). Types of tympanoplasty performed in this study included types I, III, and IV, denoting different statuses of the middle ear and ossicular defects following the removal of the cholesteatoma. Repositioning of sculptured bone grafts or prostheses was utilized in both type III and type IV tympanoplasties to reconstruct the sound conduction mechanism in the tympanic cavity, in accordance with the classification proposed by the Japan Otological Society in 2010 [18]. These tympanoplasties involved different ossicular chain assemblies and are thus further subdivided in this study as tympanoplasty type III-i (graft placing between the manubrium and the stapes head as interposition), type IV-i (graft placing between the manubrium and the footplate as interposition), type III-c (graft placing on the stapes head as columella under surface of the tympanic membrane (TM)), and type IV-c (graft placing on the footplate as columella under surface of the TM). Materials used for ossiculoplasty include autologous incus, malleus head, cortical bone, and titanium prosthesis.

### 2.4. Statistical Analysis

For descriptive analyses, we used a chi-square analysis and McNemar's test for categorical variables and a one-way analysis of variance (ANOVA) for continuous traits (with Least Significant Difference (LSD) test for post hoc comparison). Differences between the measurements of preoperative and postoperative pure-tone audiometry were tested using the paired *t*-test. Mean ± standard deviation (SD), frequency, and percentages were used to describe the characteristics of the study subjects. All statistical analyses were carried out using the SPSS for Windows (version 16.0; SPSS Corp., Chicago, IL, USA). *P* values below the conventional level of statistical significance (*P* < 0.05) were considered statistically significant.

## 3. Results

### 3.1. Demographics

Patients' ages ranged between 6 to 76 years. Significantly more patients were surgically treated for cholesteatoma in the age range of 21 to 40 years (37.7%) and 41 to 60 years (44.1%) than the other age groups (Table 1). We did not recruit CWD cases with a greater than 50% destruction of the EAC or cases that involved additional obliteration materials such as musculoperiosteal flaps, in addition to bone chips/paté as this study aimed to evaluate the impact of reconstruction of the EAC by mastoid exclusion/obliteration where bone chips/paté were applied as the sole material in retrograde mastoidectomy. Very few cases included in this study required meatoplasty to remove the soft tissue and cartilage of the ear, as there was no open cavity mastoid left following this unique surgical procedure.

In this study, we presented two types of reconstruction for EAC and mastoid cavity, using mastoid exclusion in 63 ears (61.8%) and mastoid obliteration in 39 ears (38.2%). In mastoid exclusion, bone chips alone or combined with paté were used to completely isolate the mastoid cavity from the middle ear, but the mastoid cavity was not totally obliterated as is done in the mastoid obliteration technique. In other words, we isolated the mastoid cavity but did not totally block its air from communicating with the tympanic cavity and the mastoid cells. We placed bone chips and bony plate fragments in an interlocking position around the aditus ad antrum region, which not only rebuilds the posterior bony external canal wall but also provides greater strength in order to resist further drum retraction.

Tympanoplasty type I was performed in 23 of 102 ears (22.5%), type III in 60 of 102 ears (58.9%), and type IV in 19 of 102 ears (18.7%). The mean follow-up period was 30.2 months, with a range of 18 to 72 months. In more than 71% of ears, the audiometric tests were monitored after more than 2 years (Table 1). Primary surgery constituted the majority of cases (64.7%) and the remaining 35.3% cases were surgically treated as a revision procedure.

### 3.2. Postoperative Hearing Outcomes Evaluated by Pure-Tone Average and Air-Bone Gaps

To evaluate the overall hearing outcomes after surgery, we analyzed the change in hearing thresholds obtained from AC and ABGs. As shown in Table 2, the mean four-frequency PTA via postoperative AC thresholds was 48.58 dB HL, as compared with 54.28 dB HL preoperatively (mean difference, 5.7 dB; *P* < 0.001). The mean postoperative ABG was 22.25 dB, as compared with 29.22 dB preoperatively (mean difference, 6.96 dB; *P* < 0.001). The results of these hearing assessments indicate a significant improvement in hearing gain following surgery.

### 3.3. Frequency-Specific Hearing Outcomes after Surgery

**Figure 3:**
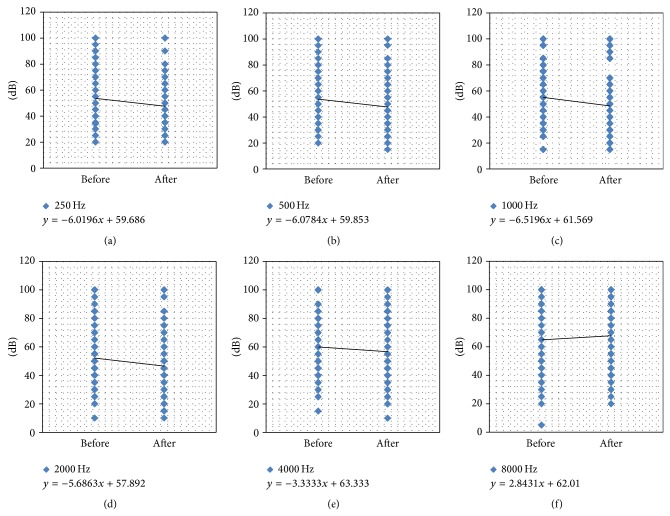
Individual audiometric outcomes in PTA. Linear regression analyses of PTA before (*x*-axis, left scatter plots) and after (*x*-axis, right scatter plots) surgery at different frequency are shown. PTA, pure-tone average.

To further investigate the postoperative changes in hearing thresholds at each octave frequency, an equation was derived through linear regression analysis of PTA before and after surgery. As demonstrated in Figure 3, a more negative slope (a decline from left to right) infers a greater postoperative improvement in hearing. Conversely, a positive slope implicates deterioration in postoperative hearing. Negative slopes were observed at 250, 500, 1000, 2000, and 4000 Hz. As the octave frequency increased from 250 to 1000 Hz, the gradient of the slope increased and was further removed from zero (−6.02 at 250 Hz, −6.08 at 500 Hz, and −6.52 at 1000 Hz, resp.). The slopes maintained negative values of −5.86 at 2000 Hz and −3.33 at 4000 Hz. However, a positive value of 2.84 was observed at 8000 Hz. These data indicate that patients who received surgery often experienced a hearing gain at low and middle frequencies, but their hearing deteriorated at a frequency of 8000 Hz.

### 3.4. Degree of Hearing Loss before and after Surgery

In addition to investigating postoperative hearing gain (Table 2), we made further investigations into the changes in hearing level following surgery. Based on the classification of degree of hearing loss as defined by Clark [17], the comparison of the change in degree of hearing loss among 102 cases is shown in Table 3. The McNemar test was used to determine the incidence rates of the degree of change in hearing loss following surgery. The improvement percentages for moderate, severe, and profound hearing loss were 38.8% (19 out of 49), 40% (6 out of 15), and 25% (2 out of 8), respectively. The results indicate that there is a statistically significant difference (*P* = 0.04).

From a total of 30 cases of mild preoperative hearing loss, 22 of these (73.3%) retained the same degree of hearing loss postoperatively, and only 8 cases (26.7%) declined to moderate hearing loss. From 49 cases of moderate preoperative hearing loss, 19 cases (38.8%) improved to mild hearing loss, 29 cases (59.2%) remained in moderate hearing loss, and 1 case (2.0%) declined to severe hearing loss postoperatively. From 15 cases of severe preoperative hearing loss, 6 cases (40.0%) improved to moderate hearing loss, 6 cases (40.0%) remained in severe hearing loss, and 3 cases (20.0%) declined to profound hearing loss. Of those with profound hearing loss (*n* = 8), only 2 cases (25.0%) improved to severe or moderate hearing loss and the majority remained in profound hearing loss postoperatively.

These results suggest that patients with mild preoperative hearing loss have a high chance of preserving their hearing status following this surgery. Patients with a moderate degree of preoperative hearing loss may have a 38.8% chance of improving their hearing degree and only a 2% chance of their hearing further deteriorating following surgery. However, there is only a 25% chance that patients with profound preoperative hearing loss will experience an improvement and only a minimal chance for their hearing to postoperatively improve to a mild degree.

### 3.5. Hearing Outcomes following Different Types of Tympanoplasty

To determine the differences in results between the various types of tympanoplasty, postoperative hearing gain in AC and ABG at each octave frequency is given in Table 4. A one-way analysis of variance (ANOVA) was performed to evaluate postoperative hearing gain between types of tympanoplasty and the results showed no statistically significant difference between surgery types for hearing gain in AC at 4000 and 8000 Hz. However, a statistically significant difference was observed at 2000 Hz (*P* = 0.03) and a borderline significant trend was found at 250 (*P* = 0.13), 500 (*P* = 0.11), and 1000 Hz (*P* = 0.08), respectively. Upon correlating the postoperative ABG gains in the different types of tympanoplasty, a distinct trend toward significance was shown at 2000 Hz (*P* = 0.07), and a borderline significant trend was shown at 1000 (*P* = 0.13). There was no statistically significant difference at 500 and 4000 Hz.

To assess for significant differences in hearing gain between types of tympanoplasty, the post hoc test indicated that type III-i was better than type I or type IV-c in AC gain across frequencies between 250 and 2000 Hz. Similarly, in ABG gain, the post hoc test revealed that type III-i was better than type IV-i at 1000 Hz, and type III-i was better than type I or type IV-c at 2000 Hz.

Analysis of hearing gain for individual ears at different frequencies, for each type of tympanoplasty, was conducted by paired *t*-test. The results demonstrated statistically significant differences (*P* < 0.001) in AC gain in type III-i from 250 to 2000 Hz, and from 500 to 2000 Hz for ABG gain. As shown in Table 4, statistically significant differences in hearing gain can be found at various frequencies in type III-c (*P* = 0.01 and *P* = 0.03) and type I (*P* = 0.04) but not in type IV-i. Taken together, we may conclude that patients benefit from tympanoplasty surgery by exhibiting different gains at various frequencies. The gain increased markedly in type III-i, followed by type III-c, type I, and type IV-c.

### 3.6. Surgical Outcomes

**Figure 4:**
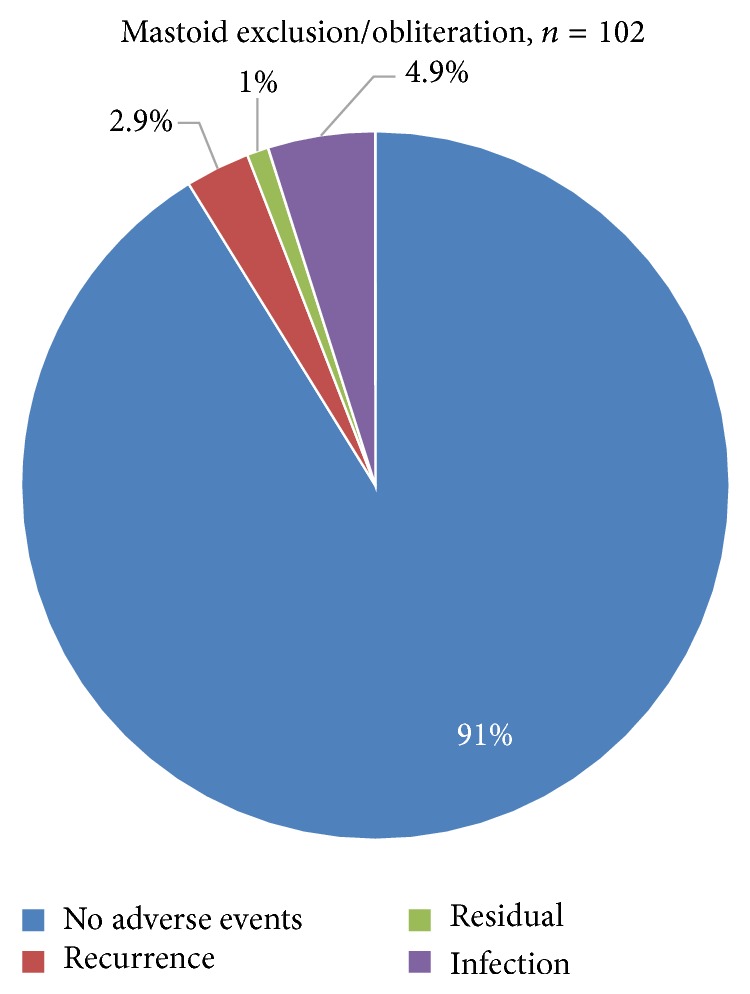
Surgical results in mastoid obliteration/exclusion.

The overall rate of postoperative adverse events was 8.8% (9 of 102). In total, five cases (4.9%) had wound infection, three cases (2.9%) had recurrence, and one case (1%) had residual disease (Figure 4). There were no significant differences between the mastoid exclusion and obliteration groups concerning the rate of recurrence, residual disease, and infection (*P* = 0.213) (Table 5). In the mastoid exclusion group, recurrence was the most common complication and occurred in three cases (4.8%), followed by infection in two cases (3%). The sites of recurrence were found in the epitympanum (2 cases), followed by the sinus tympani (1 case); these were observed at the 3-, 4-, and 5-year follow-ups, respectively. Meanwhile, in the mastoid obliteration group, infection was the most common complication, occurring in three cases (7.7%), followed by one case of residual disease (2.6%) that emerged within 1.5 years and developed in the epitympanum region. In total, significant postoperative ear discharge was found in five cases (4.9%); this related to poor epithelization of the fascia graft, meatal flap granulation overgrowth, and fungal infection of the EAC. All of these infections were observed at 3 weeks postoperatively after removal of the ear packing. However, they responded well to an oral broad-spectrum antibiotic associated with topical antiseptic antifungal ear drops, and occasionally, in-office local treatment for cauterization of the granulation tissue. No cases required reoperation.

## 4. Discussion

Although the idea of temporarily removing the posterior canal wall to allow for better exposure for cholesteatoma extirpation and subsequently reconstructing the canal wall is not new, there is no known perfect solution. Several modifications to the technique are still under development in an attempt to make such an approach technically feasible in terms of desired outcomes [5, 19–22].

In addition to mastoid obliteration, we offered mastoid exclusion for the purpose of reconstructing the EAC and preventing the development of a retraction pocket. This involved partially, but not totally, obliterating the mastoid cavity. For this technique, the mucosal residues do not need to be removed, as for mastoid obliteration. It is worth emphasizing that no more than 50% of the length of the posterior canal wall was partially removed and should be preserved as much as possible during the retrograde mastoidectomy. Otherwise, the reconstruction becomes more difficult for the reasons pointed out by Dornhoffer [5, 23] and Hinohira et al. [24].

Regardless of whether matter mastoid exclusion or obliteration is performed, both of those procedures help reconstruct the EAC defect with minimal harvest of nearby bone chips, making the procedure easy. We believe that an ideal procedure should be simple and quick. This is the distinguishing feature of the unique technique proposed in this study.

For mastoid exclusion, we placed bone chips and bony plates in an interlocking position at the exposed aditus ad antrum and EAC defect regions to effectively close off the middle ear from the attic and mastoid. Unlike similar mastoid exclusion procedures reported in previous literatures [5, 21, 22, 25], the techniques used in this study did not involve any other materials as a support but were still able to enhance and prop up the reconstructed canal wall. We prefer applying such a technique in a relatively well-pneumatized mastoid cavity owing to its adequate Eustachian tube function, allowing for aeration between the middle ear and mastoid cavity to be reserved. This is in agreement with the comments from a previous study that stated that aeration of the mastoidectomy cavity is important to prevent collapse of the posterior canal wall and retraction pockets and to insure an adequate air reserve [26].

In contrast, a sclerotic mastoid cavity usually implies the coexistence of a dysfunction of the Eustachian tube and middle ear inflammation. This contributes to the suppression of mastoid air cell development from early childhood [27]. In this study, mastoid obliteration is indicated for such a condition. The goal of mastoid obliteration is to build up a seamless wall when separating the tympanic cavity and mastoid cavity, eliminating the negative pressure effect produced by mastoid mucosa and blocking the recurrence of cholesteatoma extending into the mastoid cavity [21, 22, 25]. In practice, obliteration will inevitably be performed subsequent to the attic obliteration and posterior wall reconstruction in a small sclerotic mastoid cavity. Although an open mastoid cavity, when connected to tympanic cavity, may possess the air reservoir function that prevents a sudden drop of air pressure in the middle ear cavity [28], there is evidence that normal aeration of the middle ear can be observed when the mastoid cavity is obliterated [25, 29].

In this study, both AC and ABG values improved significantly after surgery (Table 2), indicating that patients can generally benefit from this type of operation. Furthermore, we observed that patients with a lower level of deterioration in their preoperative hearing had a higher chance of postoperatively preserving relatively good hearing, which reflects the great advantage of employing surgical techniques when it comes to accomplishing both the eradication of cholesteatoma and the preservation of function. A similar finding was observed when comparing serviceable hearing outcomes (PTA ≤ 20 dB) before and after surgery (data not shown). More than 83% of cases of preoperative serviceable hearing remained this way postoperatively. Since hearing preservation is one of the greatest concerns in patients with mild preoperative hearing loss, greater care has been applied to hearing preservation surgical techniques. The results shown in our study are encouraging and help to assure the patient of a favorable benefit to risk ratio for postoperative hearing outcomes.

In comparing the effect of the types of tympanoplasty on postoperative hearing gain, type III-i was found to be superior to other types (Table 4). This highlights the impact of the suprastructure of the stapes on hearing outcomes [30–34]. Chang and Chen [32] previously reported that 67.8% of patients with stapes suprastructure versus 23.7% of patients without stapes suprastructure reached their ABG within 20 dB, postoperatively. In addition, the position of prosthesis placement on the footplate can also influence the hearing outcome in type IV tympanoplasty. Placement on the center footplate site has the best result, followed by the anterior and posterior footplate sites [18]. This suggests that the structural mechanism for sound transmission in type IV tympanoplasty is less optimal compared with the natural structure of intact stapes.

In our report, the maximum air gain was at 1000 Hz, which then decreased as the frequencies increased. There was a limited hearing gain above 4000 Hz and an adverse impact at 8000 Hz. It is also noteworthy that ossiculoplasty had the greatest impact on hearing gain at low and mid frequencies (from 250 to 2000 Hz), but only a limited impact on frequencies above 4000 Hz, as shown in Table 5. This result is in agreement with previous reports [18, 35, 36]. In 1997, Merchant et al. [36] analyzed the middle ear mechanism and demonstrated that the middle ear would reach its maximum gain of 25 dB at around 1000 Hz and then decrease by about 6 dB per octave at frequencies above 1000 Hz. Choi et al. [35] demonstrated a similar conclusion that hearing gain was primarily achieved in low and mid frequencies, and most cases showed unfavorable postoperative hearing outcomes in high frequencies.

Many authors performed staged operation as a second-look procedure and staged ossiculoplasty [6, 22, 24]. We achieved successful hearing outcomes from a single-stage ossiculoplasty in a majority of patients. Since patients might be reluctant to consider a staged operation, unless there is cholesteatoma recurrence and related complications, achieving successful hearing outcomes from a one-stage operation is important in meeting patients' expectations.

With regard to the adverse events of surgery, no significant differences could be demonstrated between the two methods. In research on recurrent and residual cholesteatoma, many authors have determined that epitympanoplasty or epitympanic obliteration is crucial in preventing retraction pocket formation and lowering the residual rate [6, 21, 22, 24, 25]. In developing the techniques, we encountered such complications in the initial stages, and these resulted in inadequate epitympanic obliteration. In addition, the relatively high recurrence rate in the mastoid exclusion group (3 out of 63 ears) compared to the obliteration group (0 in 39 ears) might be blamed on the connection between tympanic and mastoid cavities in an unexpected situation of poor Eustachian tube function. Residual cholesteatoma mainly involved the attic and the retrotympanum [37, 38], as shown in our study, which may be due to insufficient resection in a defective exposure, or to a very fine epidermal matrix requiring nuanced operative management.

Although infection became the leading cause of complications and a total of five cases were associated with postoperative wound infections, our techniques are relatively safe compared with those in previous studies [6, 39–41]. We believe that the use of a rotated temporalis musculofascial flap, as demonstrated in this study, made the reconstructed wall smooth and also provided good vascularization to facilitate bony graft survival, which is worthwhile in preventing subsequent infection and retraction pocket formation [42, 43].

The limitations of our study include that the hearing assessment data were obtained from different durations of follow-up, although in more than 71% of ears the audiometric tests were monitored for more than 2 years. Moreover, a second-look procedure was lacking; such a procedure has been highly recommended in CWD for a high incidence of residual cholesteatoma. This raises the question of whether the retrograde mastoidectomy technique necessitates a planned second-stage operation or whether it could be substituted for observation. In fact, in our series, recurrence was never observed in the obliterated/excluded mastoid cavity, and no cases required mastoid obliteration/exclusion to be taken down. Furthermore, in six cases, a two-stage ossiculoplasty was performed, and no residual cholesteatoma was found (data not shown). Since the recurrence rates are related to the length of follow-up [33, 44], the long-term outcomes of mastoid exclusion/obliteration should be examined in subsequent studies.

## 5. Conclusions

Retrograde tympanomastoidectomy in conjunction with canal wall reconstruction, using mastoid exclusion/obliteration, provides good hearing outcomes with low complication and recidivism rates. Postoperative hearing gains following successful procedures were largely observed in low and mid frequencies, rather than in frequencies above 4000 Hz. In retrograde tympanomastoidectomy, a mastoid exclusion technique can be substituted for mastoid obliteration in patients with a relatively well-pneumatized mastoid cavity.

## Figures and Tables

**Table 1:** Summary of the characteristics of 102 ears with cholesteatoma that underwent retrograde tympanomastoidectomy.

Categories	*n* = 102	
	Number (%) of ears	
Reconstruction by		
Mastoid exclusion	63	(61.8)
Mastoid obliteration	39	(38.2)
Total	**102**	**(100)**
Types of tympanoplasty		
I	23	(22.5)
III-c	11	(10.8)
III-i	49	(48.1)
IV-c	12	(11.8)
IV-i	7	(6.9)
Total	**102**	**(100)**
Follow-up periods		
1-2 years	29	(28.2)
2-3 years	48	(47.1)
>3 years	25	(24.5)
Total	**102**	**(100)**
Age distribution		
<10 years	3	(2.9)
10–20 years	9	(8.8)
21–40 years	38	(37.3)
41–60 years	45	(44.1)
>60 years	7	(6.9)
Total	** 102**	** (100)**
Reconstruction performed as		
Primary surgery	66	(64.7)
Revision surgery	36	(35.3)
Total	**102**	**(100)**

i: interposition; c: columella.

**Table 2:** Audiometric results before and after surgery.

Audiometric results	*n* = 102		
	Mean	SD	*p* value
PTA before surgery, dB HL	54.28	20.54	
PTA after surgery, dB HL	48.58	21.58	
^*^PTA changes, dB = before surgery − after surgery	5.70	13.60	<0.001

ABG before surgery, dB HL	29.22	13.04	
ABG after surgery, dB HL	22.25	11.11	
^*^ABG changes, dB = before surgery − after surgery	6.96	10.97	<0.001

PTA: pure-tone average; ABG: air-bone gap; ^*^paired *t*-test.

**Table 3:** Postoperative changes in degree of hearing loss (*n* = 102).

Degree of hearing loss^b^	PTA^a^ before surgery				*P* value
	Mild hearing loss (*n* = 30)	Moderate hearing loss (*n* = 49)	Severe hearing loss (*n* = 15)	Profound hearing loss (*n* = 8)	
PTA after surgery					
Mild hearing loss (*n* = 41)	22	19	0	0	0.04
Moderate hearing loss (*n* = 44)	8	29	6	1	
Severe hearing loss (*n* = 8)	0	1	6	1	
Profound hearing loss (*n* = 9)	0	0	3	6	

^a^PTA: pure-tone average.

^b^The degree of hearing loss was defined by the source of Clark, (1981) [17] (i.e., mild hearing loss: ≤40 dB, moderate hearing loss: 41–70 dB, severe hearing loss: 71–90 dB, profound hearing loss: ≥91 dB).

**Table 4:** Comparison of hearing gains at various frequencies with respect to the type of tympanoplasty that was performed.

Hearing gain	Type I (*n* = 23)		*P* ^a^	Type III-c (*n* = 11)		*P*	Type III-i (*n* = 49)		*P*	Type IV-c (*n* = 12)		*P*	Type IV-i (*n* = 7)		*P*	*p* value^b^	Post hoc
	Mean	SD		Mean	SD		Mean	SD		Mean	SD		Mean	SD			
AC^c^																	
250 Hz	1.09	13.65	0.71	7.27	9.32	0.03	8.96	13.15	<0.001	4.58	12.70	0.24	2.14	4.88	0.29	0.13	III-i > I
500 Hz	−0.22	13.94	0.94	8.64	12.86	0.05	9.08	14.85	<0.001	4.58	10.33	0.15	4.29	10.18	0.31	0.11	III-i > I
1000 Hz	3.04	15.65	0.36	11.36	14.33	0.03	9.59	15.64	<0.001	−0.42	9.88	0.89	0.71	5.35	0.74	0.08	III-i > IV-c
2000 Hz	0.65	16.60	0.85	8.64	11.42	0.03	10.00	16.65	<0.001	−3.75	13.51	0.36	3.57	9.88	0.38	0.03	III-i > I, IV-c
4000 Hz	−0.22	16.89	0.95	3.64	11.20	0.31	4.59	15.97	0.05	5.00	16.65	0.32	2.86	7.56	0.36	0.79	—
8000 Hz	−6.09	18.34	0.13	−8.18	14.37	0.09	0.31	15.46	0.89	−3.33	8.62	0.21	−5.00	12.25	0.32	0.34	—
ABG^d^																	
500 Hz	5.00	13.90	0.10	12.27	13.30	0.01	8.57	13.19	<0.001	6.25	10.03	0.05	3.57	9.45	0.36	0.48	—
1000 Hz	6.96	15.13	0.04	12.73	13.11	0.01	8.98	13.07	<0.001	2.08	8.91	0.44	−0.71	8.86	0.84	0.13	III-c > IV-i
2000 Hz	3.48	14.96	0.28	9.64	10.32	0.01	10.00	10.85	<0.001	1.67	11.93	0.64	1.43	15.20	0.81	0.07	III-i > I, IV-c
4000 Hz	4.78	16.13	0.17	7.73	12.12	0.06	4.90	11.92	0.01	5.00	12.79	0.20	1.43	8.99	0.69	0.91	—

^a^ *P* represented as significant level of paired *t*-test.

^b^ *p* value represented as significant level of ANOVA test, post hoc by LSD.

^c^AC: air conduction.

^d^ABG: air-bone gap.

**Table 5:** The association between the type of ear canal reconstruction and surgical outcomes.

	Exclusion (*n* = 63)	Obliteration (*n* = 39)	*p* value
Adverse events			0.213
None	58	35
With recurrence	3	0
With residual	0	1
With infection	2	3
